# Early Aortic Valve Replacement Versus Conservative Management: A Meta-Analysis of Randomized Controlled Trials

**DOI:** 10.1016/j.shj.2025.100461

**Published:** 2025-03-19

**Authors:** Tayyab Shah, Yousuf Shah, Alexandra J. Lansky, Jay Giri, Alexander Fanaroff, Ashwin Nathan

**Affiliations:** a Division of Cardiovascular Medicine, Hospital of the University of Pennsylvania, Philadelphia, Pennsylvania, USA; b Department of Internal Medicine, Strong Memorial Hospital, Rochester, New York, USA; c Division of Cardiology, Yale School of Medicine, New Haven, Connecticut, USA; d Barts Heart Centre, London and Queen Mary University of London, London, UK

**Keywords:** Aortic stenosis, Aortic valve replacement, Asymptomatic, Early conservative

## Abstract

• Early aortic valve replacement before the development of severe symptomatic aortic stenosis is not associated with a benefit for most hard endpoints including mortality and myocardial infarction but is associated with a benefit in stroke.
• The mechanism for the potential benefit of early aortic valve replacement on stroke remains unclear.
• Younger patients and women may derive a mortality benefit from early aortic valve replacement, but this warrants further study.

Management of aortic stenosis (AS) typically involves surveillance until severe, symptomatic AS develops, at which point aortic valve replacement (AVR) is recommended. However, recent trials suggest that earlier transcatheter AVR or surgical AVR (TAVR or SAVR)—performed before patients reach symptomatic severe AS—may improve outcomes. We conducted a meta-analysis to synthesize data from recent randomized controlled trials (RCTs) comparing early AVR with conservative management.

This meta-analysis followed the Preferred Reporting Items for Systematic Reviews and Meta-Analyses (PRISMA) guidelines and was registered in PROSPERO (CRD42024609683). A search of the PubMed and clinicaltrials.gov databases identified RCTs comparing early TAVR/SAVR with conservative management in patients with asymptomatic moderate or severe AS published between January 1, 2016, and November 4, 2024, using the terms early, asymptomatic, aortic valve replacement, TAVR, SAVR, and AS. Two reviewers (T.S. and Y.S.) independently screened titles, abstracts, and full texts for eligibility and extracted data with disagreements resolved by consensus. The primary endpoint was all-cause mortality, and secondary endpoints included myocardial infarction (MI), stroke, periprocedural stroke, postprocedural atrial fibrillation (AF), and postprocedural permanent pacemaker (PPM) implantation. Heterogeneity was assessed using the I^2^ statistic, and random-effects models were used to aggregate relative risks (RRs) for each endpoint assessed at the longest available follow-up. Meta-regression analyses using trial-level covariates examined potential effect modifiers for mortality and stroke, including mean age, proportion females, intervention type (TAVR/SAVR), and proportion of patients in the control arm who received AVR.

Our search yielded 49 manuscripts and 19 registered trials, of which 5 RCTs (1605 patients) met the eligibility criteria.^1^, ^2^, ^3^, ^4^, ^5^ Two trials (Randomized Comparison of Early Surgery Versus Conventional Treatment in Very Severe Aortic Stenosis [RECOVERY] and Aortic Valve Replacement Versus Conservative Treatment in Asymptomatic Severe Aortic Stenosis [AVATAR]) evaluated SAVR in asymptomatic severe AS,^1^^,^^2^ 1 trial (Evaluation of Transcatheter Aortic Valve Replacement Compared to Surveillance for Patients With Asymptomatic Severe Aortic Stenosis [EARLY TAVR]) evaluated TAVR in asymptomatic severe AS,^3^ 1 (Transcatheter Aortic Valve Replacement to UNload the Left Ventricle in Patients With ADvanced Heart Failure [TAVR UNLOAD]) evaluated TAVR in moderate AS with heart failure,^4^ and 1 (Early Valve Replacement Guided by Biomarkers of Left Ventricular Decompensation in Asymptomatic Patients with Severe Aortic Stenosis [EVOLVED]) evaluated both SAVR and TAVR (75 and 25%, respectively) in severe AS.^5^ Median follow-up across studies ranged from 1.9 to 6.2 years.

The pooled analysis for mortality did not find a significant benefit to early AVR compared to conservative management (RR: 0.77; 95% CI: 0.54-1.11; *p* = 0.16) (Figure 1). Early AVR was associated with a significantly lower risk of any stroke (RR: 0.59; 95% CI: 0.39-0.90; *p* = 0.01) but had no significant effect on MI (RR: 0.46; 95% CI: 0.09-2.37; *p* = 0.35). There was also no significant benefit regarding periprocedural stroke (RR: 0.48; 95% CI: 0.17 - 1.31; *p* = 0.15), postprocedural AF (RR: 1.43; 95% CI: 0.87-2.36; *p* = 0.16), or postprocedural PPM implantation (RR: 0.87; 95% CI: 0.34-2.27; *p* = 0.78) among patients who received an AVR. There was no significant heterogeneity across trials for any endpoint. Results were consistent when excluding the Transcatheter Aortic Valve Replacement to UNload the Left Ventricle in Patients With ADvanced Heart Failure (TAVR UNLOAD) trial. Of the tested effect modifiers, meta-regression identified 3 significant effect modifiers for mortality but none for stroke, with lower mean age (beta = 0.072, *p* = 0.007), higher proportion of women (beta = −0.034, *p* = 0.007), and SAVR relative to TAVR (beta = −0.57, *p* = 0.033) associated with improved mortality. Multiple testing was not adjusted for.

**Figure 1:**
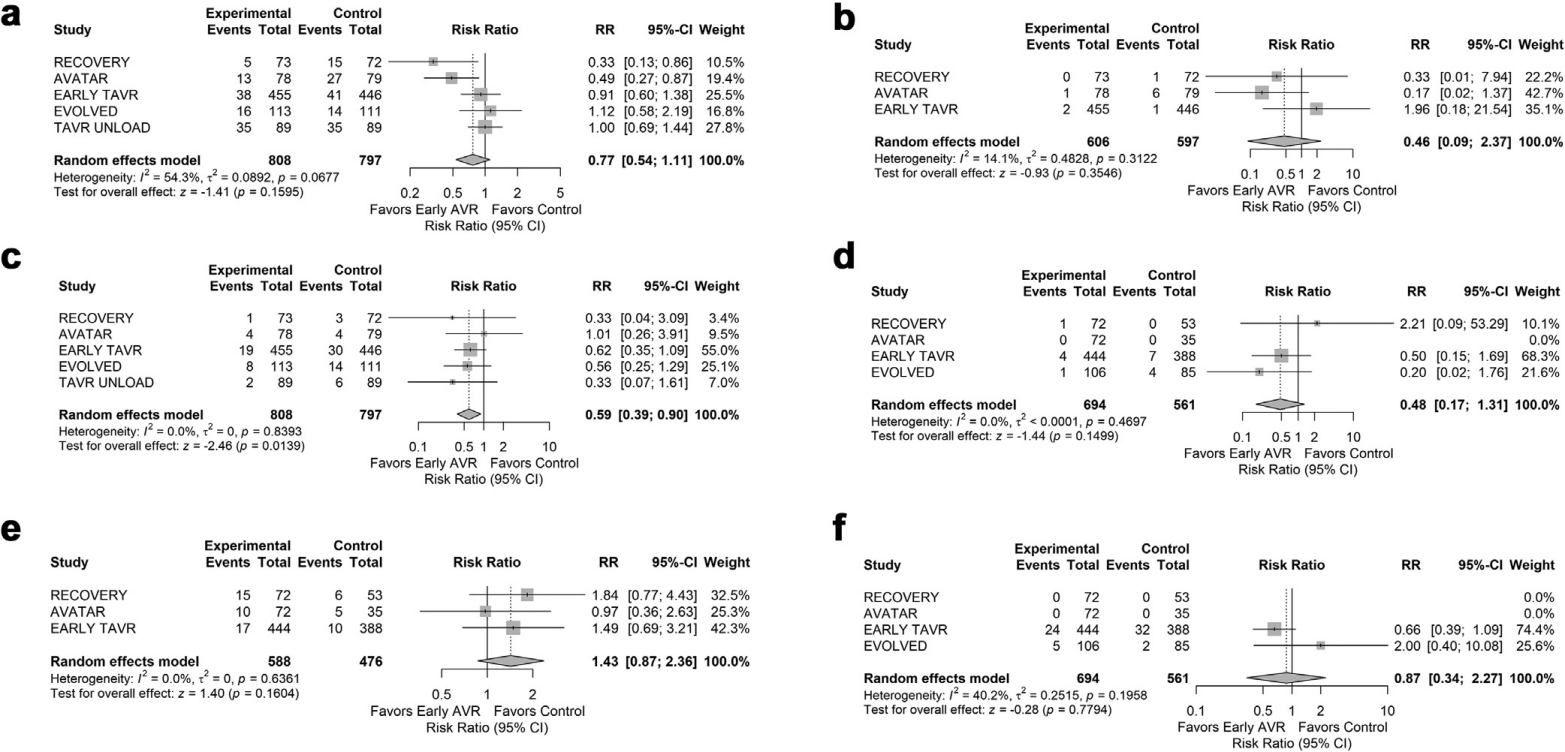
Forest plots for (a) mortality, (b) myocardial infarction, (c) stroke, (d) periprocedural stroke, (e) postprocedural atrial fibrillation, (f) permanent pacemaker implantation. Continuity correction using a default value of 0.5 was used for rows where one trial had a zero event rate. Abbreviations: AVATAR, Aortic Valve Replacement Versus Conservative Treatment in Asymptomatic Severe Aortic Stenosis; AVR, aortic valve replacement; CI, confidence interval; EARLY TAVR, Evaluation of Transcatheter Aortic Valve Replacement Compared to Surveillance for Patients With Asymptomatic Severe Aortic Stenosis; EVOLVED, Early Valve Replacement Guided by Biomarkers of Left Ventricular Decompensation in Asymptomatic Patients with Severe Aortic Stenosis; RECOVERY, Randomized Comparison of Early Surgery Versus Conventional Treatment in Very Severe Aortic Stenosis; RR, relative risk; TAVR UNLOAD, Transcatheter Aortic Valve Replacement to UNload the Left Ventricle in Patients With ADvanced Heart Failure.

This meta-analysis provides a contemporary synthesis of evidence on early AVR in patients without symptomatic severe AS. Similar to a recent meta-analysis that did not include the TAVR UNLOAD trial,^6^ we found no mortality benefit with early AVR but did find that early AVR was associated with a substantially reduced risk of stroke. We also analyzed other hard outcomes including MI and postprocedural PPM implantation and did not identify any benefit to early AVR. Unlike the prior meta-analysis, our analysis looks more in depth at trial-level factors that could affect the impact of early AVR on mortality. Indeed, the meta-regression suggests that younger patients, females, and those receiving SAVR may have a mortality benefit from early AVR. It is difficult to completely distinguish whether it was SAVR or young age driving the potential mortality benefit given the 2 SAVR-only trials were also the ones that enrolled the youngest patients (mean ages 64 and 67 years vs. 73-77 years). Still, it should be noted that the EVOLVED trial which enrolled older patients (mean age 73 years) and used SAVR in 75% of patients did not find a mortality benefit, while the 2 SAVR-only trials did, *and* mean age was more strongly associated with mortality effect size in the meta-regression relative to the effect of SAVR. This at least suggests that mean age may be driving differing results on mortality between the early SAVR trials and the more recent early TAVR trials. Finally, the potential benefit among females is also plausible given it is well-documented that women with symptomatic severe AS have longer delays to treatment, which may be mitigated with early AVR.

This meta-analysis also looks more in depth at the potential mechanism behind early AVR being associated with a reduced risk of stroke than the prior analysis. For one, it could be related to the fact that earlier AVR of presumably less calcified valves may reduce periprocedural embolic events—indeed periprocedural strokes are numerically lower in the early AVR arm. It does not appear that postprocedural AF occurs less frequently with early AVR; however, we did not have data on whether the longer exposure to higher aortic valve gradients resulted in greater AF burden among patients in the conservative arms. We also did not find that earlier AVR reduced future procedure burden (i.e., PPM implantation or revascularization for future MI), which also could have potentially reduced the stroke risk inherent to these procedures. It is possible that routine antithrombotic usage post earlier AVR may provide long-term risk reduction. Unfortunately, this could not be tested given only 1 trial reported on any antithrombotic usage. Thus, the mechanism of reduced stroke risk seen with early AVR remains unclear.

This study is limited by significant differences in trial participants (moderate vs severe AS) and interventions (surgical vs transcatheter). However, all trials were included in this meta-analysis because they addressed the same clinical question: does earlier AVR improve clinical outcomes before AS causes irreversible damage? Additionally, there was no significant heterogeneity for any outcome, and despite trial-level differences, most patients in the control arms received an AVR within 1-2 years, even those in the TAVR UNLOAD study, demonstrating that there were similar risk patients across the trials. Additionally, we addressed some of these differences with meta-regression and the results were consistent when the TAVR UNLOAD trial was excluded. In fact, the inclusion of all 5 trials with a broad and varying patient population allowed for identification of potential effect modifiers. Overall, these results are hypothesis generating and warrant further study in RCTs.

## Ethics Statement

This study used publicly available trial-level data without any identifiable patient information and thus did not require ethical approval.

## Funding

The authors have no funding to report.

## Disclosure Statement

A. Nathan received speaking fees and research support from 10.13039/100006520Edwards Lifesciences. The other authors had no conflicts to declare.

## References

[1] Kang D.H., Park S.J., Lee S.A. (2020). Early surgery or conservative care for asymptomatic aortic stenosis. N Engl J Med.

[2] Banovic M., Putnik S., Da Costa B.R. (2024). Aortic valve replacement vs. conservative treatment in asymptomatic severe aortic stenosis: long-term follow-up of the AVATAR trial. Eur Heart J.

[3] Généreux P., Schwartz A., Oldemeyer J.B. (2025). Transcatheter aortic-valve replacement for asymptomatic severe aortic stenosis. N Engl J Med.

[4] Van Mieghem N.M., Elmariah S., Spitzer E. (2025). Transcatheter aortic valve replacement in patients with Systolic heart failure and moderate aortic stenosis: TAVR UNLOAD. J Am Coll Cardiol.

[5] Loganath K., Craig N.J., Everett R.J. (2025). Early intervention in patients with asymptomatic severe aortic stenosis and myocardial fibrosis: the EVOLVED randomized clinical trial. JAMA.

[6] Généreux P., Banovic M., Kang D.-H. (2025). Aortic valve replacement vs clinical surveillance in asymptomatic severe aortic stenosis: a systematic review and meta-analysis. J Am Coll Cardiol.

